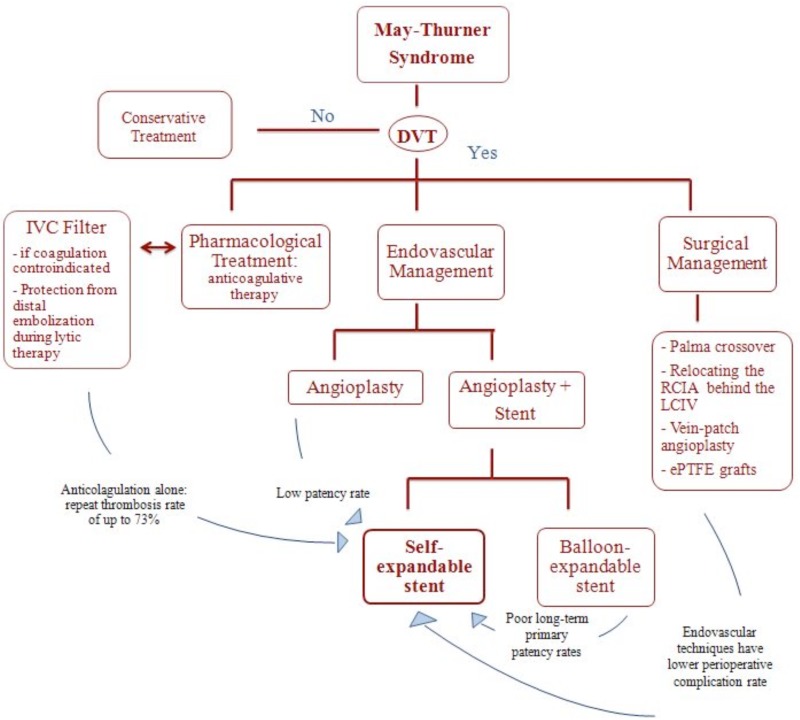# What the young physician should know about May-Thurner syndrome

**Published:** 2014-09-01

**Authors:** Donatella Narese, Umberto Marcello Bracale, Gaetano Vitale, Massimo Porcellini, Massimo Midiri, Giancarlo Bracale

**Affiliations:** 1 Department of Radiology D.I.B.I.M.E.F., “P. Giaccone” University Hospital, Palermo, Italy; 2 Vascular and Endovascular Surgery, Federico II University of Naples, Italy; 3 Vascular and Endovascular Surgery, University of Salerno Italy

**Keywords:** May-Thurner Syndrome, DVT, endovascular treatment, thrombectomy, medical education

## Abstract

May-Thurner syndrome (MTS) is an anatomically variable condition resulting in compression of the left common iliac vein between the right common iliac artery and the underlying spine with subsequent development of a left deep vein thrombosis (DVT). Although this syndrome is rare, its true prevalence is likely underestimated. Mainly, clinical symptoms and signs include, but are not limited to, pain, swelling, venous stasis ulcers, skin pigmentation changes and post-thrombotic syndrome. Correct treatment is not well established and is based on clinical presentation. Staged thrombolysis with/without prophylactic retrievable inferior vena cava filter placement followed by angioplasty/stenting of the left iliac vein appears to be the best option in MTS patients with extensive DVT. The aim of this review is to present in a simple and didactic form all variable clinical presentations of MTS and to outline possible management within the current guidelines.

## I. INTRODUCTION

**Fig. 1:**
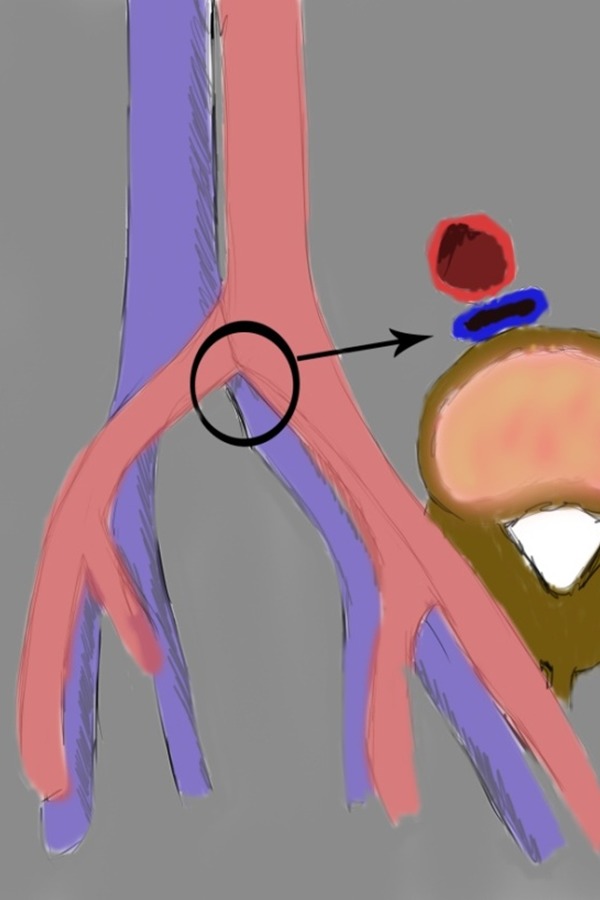
drawing showing left common iliac vein compression by right common iliac artery (morphologic type I).

May-Thurner Syndrome (MTS), otherwise known as iliac vein compression syndrome [1], is an anatomically variable condition of the left common iliac vein (LCIV) with outflow obstruction caused by the right common iliac artery compression against the lower lumbar vertebrae (Fig. 1). Right-sided MTS cases are definitively more rare but have also been reported [2,3] in the literature. The majority of patients with right-sided MTS are male which may be due to the shape of the male pelvis. Although numerous articles have described the left-sided and female predominance of MTS, no correlation has been found between right-sided MTS and male gender. Some Authors postulate that the conical shape of the male pelvis may predispose the right iliac vein to compression by the iliac artery. However Virchow was the first who described this condition in 1851 and noted that deep vein thrombosis (DVT) was five times more likely to occur in the left lower limb [4]. In 1906 Mcmurrich proposed a congenital aetiology for LCVI obstruction [5] while Ehrich and Krumbhaar postulated, instead, a degenerative change of a venous valve at or near the origin of the LCIV [6]. Compression of the left iliac vein with detailed anatomic description was later described by May and Thurner in 1958 [7]. Cockett and Thomas also reported this condition in 1965, illustrating the relation between iliac vein compression and post-thrombotic syndrome and for this reason it is also known as “Cockett syndrome” [1]. Compression of the left common iliac vein by the right common iliac artery is a frequent anatomic variant observed in 22–32% of cadavers [8]. Most MTS patients throughout the course of their lives have no symptoms and therefore no treatment is required. Some Authors have proposed using the term “May-Thurner anatomy” in patients without hemodynamic significance and reserve the term “May-Thurner syndrome” for cases with compromised venous flow [9].

## II. METHODOLOGY

### Clinical Manifestations

MTS usually presents in the second or third decade of life and is more common in women. The majority of these patients are asymptomatic. Once symptoms develop, patients can present during the acute or chronic MTS phase. Patients with acute manifestations most frequently (18–49% of cases) present with unilateral left lower LCIV thrombosis, swelling and pain [10]. Prolonged immobilization, dehydration, multiple pregnancies, the postpartum period, contraceptive therapy, surgical intervention for gynaecologic [11] and abdominal pathologic conditions [12] or febrile illness, including pneumonia and pleurisy [13,14,15], have been described as the main risk factors.

Acute MTS can be life-threatening if there is a spontaneous retroperitoneal hematoma associated with iliac vein rupture [16,17] or an associated pulmonary embolism (PE). However, it should be pointed out that these patients rarely present with PE probably because ilio-caval compression may offer protection by trapping large emboli [18]. Chronic MTS is the result of long-term venous hypertension and is characterized by chronic venous insufficiency (CVI), varicose veins, lower-extremity skin pigmentation changes (lipodermatosclerosis), chronic leg pain, recurrent skin ulcers [14], phlegmasia cerulea dolens [19], or recurrent superficial venous thrombophlebitis. Other MTS presentations include cryptogenic stroke in patients with a patent foramen ovale [20,21], pelvic congestion syndrome [22], or priapism [23].

### Diagnostic techniques

Clinical information alone (history and physical examination) is insufficient for the diagnosis of MTS. A thrombophilia workup should always be performed to identify the risk factors for DVT, specifically in younger patients, and objective diagnostic testing is essential.

Non-invasive imaging modalities useful in the evaluation of MTS include color Doppler ultrasound, Computed Tomography (CT), Magnetic Resonance Imaging (MRI) and Intravascular Ultrasound (IVUS), while a useful invasive diagnostic test is contrast venography.

#### Color Doppler Ultrasound

The potential pitfalls of each of these diagnostic modalities must be recognized to avoid false interpretation. Over the years, because of its wide availability, portability and cost-effectiveness, ultrasound has been routinely used in the first-line evaluation of MTS patients, as it allows for a correct evaluation of the status of the deep veins of the extremity involved.

However the accuracy of ultrasonography in patients with MTS is not very convincing because of its lower sensitivity above the inguinal plane [24,19].

When visualization of the common iliac vein is possible, MTS may be diagnosed with trans-abdominal color Doppler ultrasound [25], but the overall sensitivity of US in visualization of the external iliac vein and common iliac vein is reportedly 79% and 47%, respectively [26]. If MTS is suspected in patients following ultrasound, cross-sectional imaging should be used to visualize the pelvic region.

In 2007 Labropoulos et al. [27] determined the following ultrasound criteria to detect a clinically substantial vein obstruction:

- Color flow demonstrates mosaic flow, indicating post-stenotic turbulence due to proximal venous stenosis;
- Pulse Doppler discloses absence of flow at the area of stenosis;
- Continuous flow (absence of phasic blood blow);
- Abnormal Valsalva response with continuous flow during Valsalva maneuver;
- A peak vein velocity ratio of >2.5 across the stenosis.

#### Computed Tomography and Magnetic Resonance Imaging

CT and MRI, as compared with ultrasonography, yield markedly higher accuracy values.

There is no consensus about specific radiological signs however the most useful findings are:

- Compression of the left common iliac vein by the right common iliac artery
- Tortuous venous collaterals crossing the pelvis to drain into the contralateral veins
- Thrombus formation.

Both CT and MRI have high sensitivity and specificity in the identification of DVT [28,29,30] of the venous compression and pelvic venous collaterals.

**Fig. 2:**
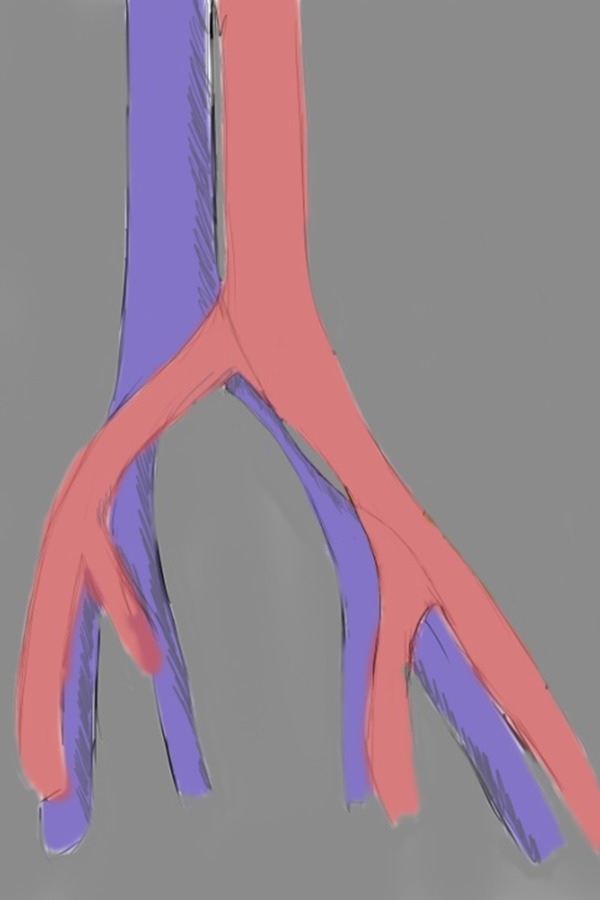
drawing showing diffuse atrophy of left common iliac vein in May-Thurner Syndrome (morphologic type II).

**Fig. 3:**
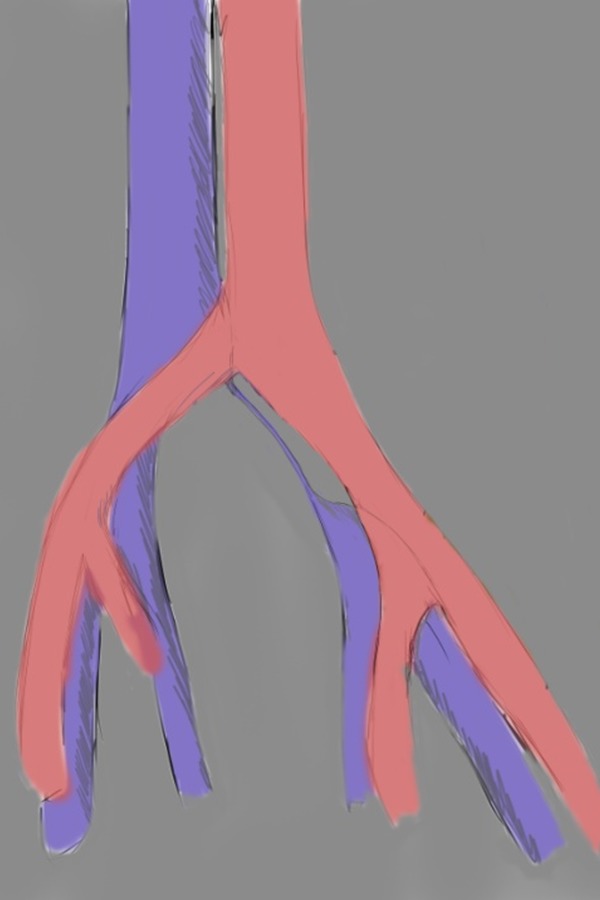
drawing showing cordlike obliteration of left common iliac vein in May-Thurner Syndrome (morphologic type III)

CT, particularly with a standardized protocol, is useful for a fast, comprehensive evaluation of the vascular system. [14]. Jeon et al. in 2010 investigated the potential role of CT venography by analyzing the morphologic features in predicting endovascular technical difficulties (fibrotic stenosis increase stent insertion) and long-term stent patency [31]. Three morphologic types of MTS have been individuated, each representing different stages of the obstruction: focal extrinsic compression, diffuse atrophy and cordlike obliteration, (Fig. 1,2,3).

The MRI, instead, permits accurate estimation of venous compression, length of obstruction and collateral vein network (Fig. 4a,b).

The recent blood pool contrast agent “gadofosveset trisodium” has increased imaging resolution, sensitivity, specificity and accuracy versus the non-enhanced MR angiography and has not been shown to cause nephrogenic systemic fibrosis [32,33].

#### Intravascular Ultrasound

Intravascular US (IVUS) has been used successfully to demonstrate iliac vein compression. It allows assessment of the intimal changes, mural abnormalities (spurs), intraluminal webs and channels in the vessel wall and can help in treatment by guiding stent placement and sizing. [34,35,36]

#### Venography

The gold standard diagnostic test is contrast venography, but it is invasive, expensive, and the contrast can cause allergic reactions or post-injection DVT. Venography with the use of trans-venous pressure measurements is considered the modality of choice for diagnosing MTS. Simultaneous pressure measurements in both external iliac veins should be obtained. Normally the pressure is 5 to 6 mm Hg at rest while during exercise it measures 7 mm Hg. To diagnose a significant stenosis, the resting pressure gradient between the 2 iliac veins should be greater than 2 mm Hg at rest and greater than 3 mm Hg during exercise. Other authors measured the pressure in the lower inferior vena cava above the site of obstruction and performed pullback pressure studies and found these methods more valid than the above measurements [37].

Advantages and disadvantages of US, CT, MRI, IVUS and contrast-enhanced venography are summarized in Tables 1.

### Treatment

Treatment dependents on the presence of DVT [40]:

- in the absence of DVT, conservative treatment is preferred;
- in the presence of DVT, the standard therapy is anticoagulation with compression bandages.

Management strategies for the treatment of MTS are summarized in Table 2.

#### Pharmacological Treatment

Historically, the treatment for MTS patients has been anticoagulation therapy. Although anticoagulation prevents clot propagation, the existing clot and the underlying mechanical compression persist. Consequently, anticoagulation alone and thrombectomy with prospective anticoagulation yielded a recurrent thrombosis in up to 73% of patients with a venous spur [41,22 ].

#### Surgical Management

**Fig. 5:**
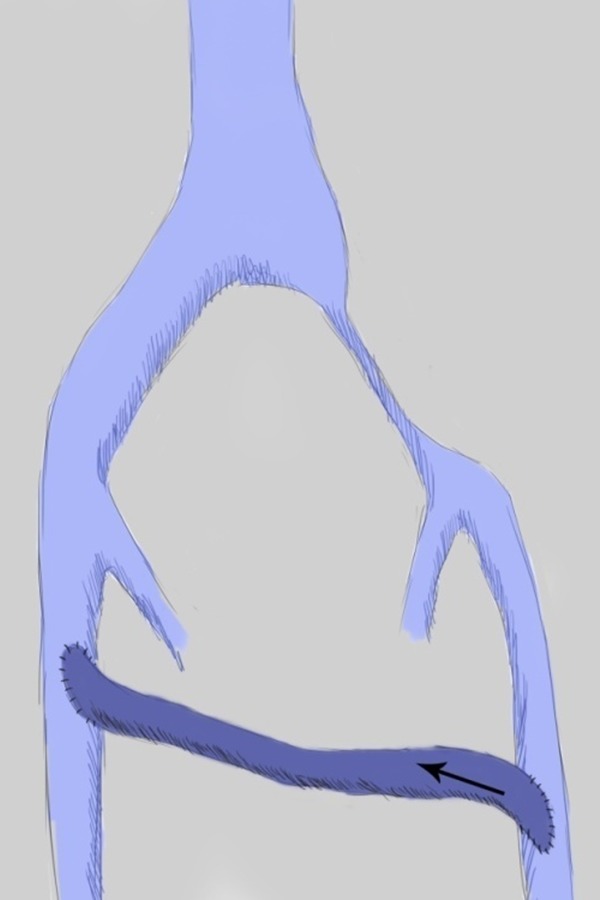
drawing showing Palma operation with autologous saphenous vein graft

Surgical management can be offered with different surgical treatment methods. (Fig. 5).

Today, MTS patients rarely undergo surgery management because endovascular techniques have been shown to have fewer operative risks [43,44]. For this reason, to date, the only indication for open surgery is failure of endovascular therapy.

#### Endovascular Management

**Fig. 6:**
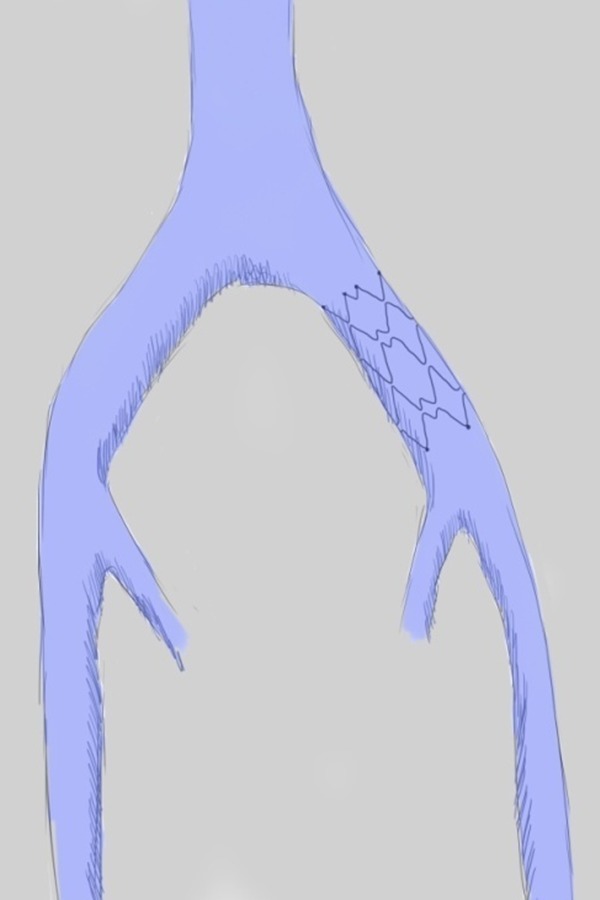
drawing showing endovascular treatment with self-expanding stent placement in left common iliac vein

Endovascular management should be the first-line treatment for MTS as demonstrated by retrospective and prospective studies [34,45,46,15,47]. Endovascular management typically begins with venography to confirm MTS and demonstrate the degree of LCIV stenosis followed by a percutaneous transluminal angioplasty (PTA) to expand the intraluminal space and finally the implantation of a self-expanding stent (Fig. 6).

Some topics remain highly controversial:

- The use of IVC filters.

If DVT is present, some practitioners suggest that a IVC filter should be placed [48,49].

However, recent Society for Vascular Survey (SVS) 2012 guidelines [50] do not recommend the placement of an IVC filter given the known long-term complications associated with IVC filters and the low risk for PE events of MTS.

- Pharmacomechanical thrombolysis versus full anticoagulation alone.

SVS guidelines suggest the benefit of early intervention with percutaneous thrombolysis [50] while the American College of Chest Physicians guidelines suggest, instead, full anticoagulation alone over regional, systemic, or mechanical thrombolysis [51].

Some respected authorities today include staged thrombolysis in the treatment of MTS, especially in the presence of extensive iliofemoral DVT [52–56]

- The superiority of a particular endovascular combination in long-term primary patency

Today it is well established that stent placement has higher patency rates than angioplasty [42] and that stainless steel stents are to be preferred to high radial force stents [57]. Brazeau et al. [58] examined various treatments but no specific treatment has yet demonstrated better outcomes.

- Type of stent

As above-mentioned, iliofemoral venous stenting is now the primary treatment option for occlusions of the venous outflow tract. Today stenting has definitively replaced the open procedures that were utilized in the past and is often performed after pharmacological or mechanical thrombus removal. The first results of ilio-femoral venous stenting were reported during the early 1990s [59]. Since then technology in stent design has much improved. Zilver vena (Cook Medical, Bloomington, IN, USA) is one of the most recent stents on the market designed specifically for this purpose. It is a self-expandable nitinol stent that provides flexibility, consistent radial force and continuous stent to vein wall apposition from end to end. Zilver vena is currently available in 14 and 16 mm diameters and 60,100 and 140 mm lengths. Most often, this type of stent is delivered from the IVC to approximately the level of the lesser trochanter. Post-dilatation is usually performed up to the nominal diameter of the stent [60].

## III. CONCLUSION

May-Thurner syndrome is a relatively rare entity and therefore knowledge of this syndrome associated with deep veins is essential. The aim of this study is to present, in a very didactic form, current management of this disorder, including diagnostic and interventional strategies. Endovascular techniques have evolved and now play a significant role in the treatment of May-Thurner syndrome. In the majority of individuals with May-Thurner syndrome it is believed that direct compression of the left iliac vein between the right iliac artery and fifth lumbar vertebrae predisposes to the formation of deep vein thrombi. The syndrome is thought to progress through 3 stages: (1) asymptomatic compression of the vein, (2) the development of intraluminal spurs (fibrous bands) at the site of compression and (3) development of deep vein thrombi. Spontaneous rupture of the LCIV is a rare occurrence.

## Figures and Tables

**Fig. 4a:**
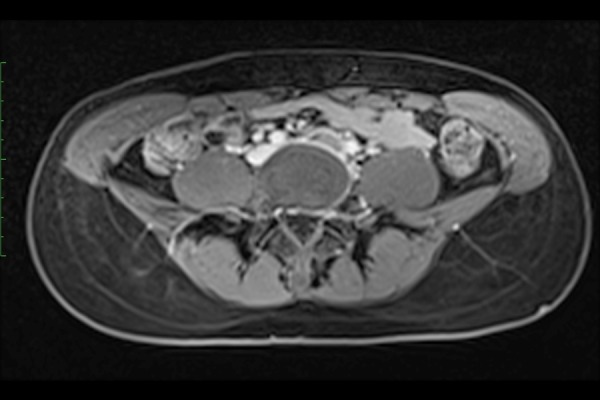
MRI of 18 year-old women showing compression and thrombotic occlusion of the left iliac vein

**Fig. 4b:**
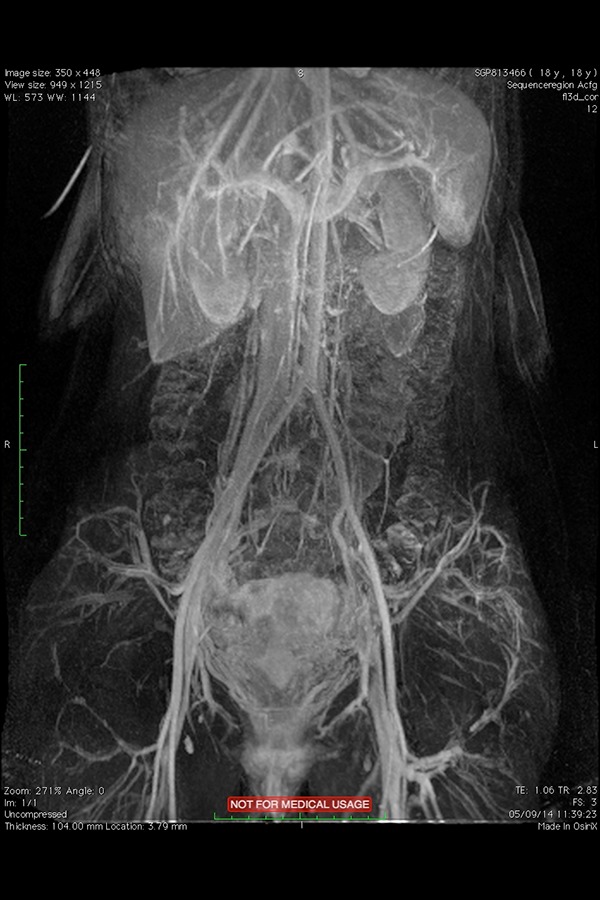
Subtracted maximum intensity projection coronal post contrast MRI showing dilated left pelvic collaterals.

**Table I:** Techniques for the diagnosis of May-Thurner syndrome

	**Advantages**	**Disadvantages**
**ULTRASOUND**	Highly sensitive and specific for proximal lower extremity DVT Least expensive imaging method Non-invasive, portable, can be done at patient’s bedside Non-ionizing radiation hazards, hence ideal for young women	Low sensitivity above the inguinal plane [25] Iliac vein thrombosis may be technically challenging to depict [25] Iliac vein compressibility may not be possible to assess [25] Operator dependent Pain, bandages, or casts may limit or prevent examination
**CT**	Non operator dependent High sensitivity and specificity MDCT allows multiplanar reconstruction of images with exquisite details More readily available and approachable compared to MRI Less expensive compared to MRI	Radiation exposure Large volume of contrast agent required to achieve opacification of veins Contrast agent toxicity Limited resolution in the pelvic region due to bony artifacts
**MRI ANGIOGRAPHY**	Non operator dependent Better contrast resolution Non-ionizing radiation hazards, hence ideal for young women Direct multiplanar imaging Better safety profile of gadolinium-based contrast agents Useful in assessing the haemodynamic significance of venous compression as it has the ability to demonstrate retrograde flow [25,38,39]	Contraindicated for patients with pacemakers and other metallic implants Not readily available High cost Metallic implant in the pelvis can create imaging artifacts
**IVUS**	Most sensitive and dynamic test to determine the degree of stenosis and to calibrate vessel before stent deployment During endovascular treatments facilitates accurate placement of a wire across the stenosis [40]	Invasive Does not yield extra-vascular information
**CATHETER VENOGRAPHY**	Allows the assessment of haemodynamic Significance Allows treatment in the same setting Pressure gradients could be measured across the compression [41]	Invasive Expansive Can cause allergic reactions or post-injection DVT Does not yield extra-vascular information

**Table II:** Flow-chart of May-Thurner syndrome management