# Errata Corrige: “What the Young Physician Should know About May-Thurner Syndrome” Transl Med Unisa. 2014 Sep 1;12:19–28

**Published:** 2016-01-31

The Authors correct list is: Narese D, Bracale U. M., Vitale G., Porcellini M., Midiri M., Bracale G. This paper was published on Pubmed listing the Authors by their first names rather than by their surnames.

